# Study on the pathogenicity, immunogenicity, endogenous development and drug sensitivity of *Eimeria kongi*

**DOI:** 10.3389/fvets.2023.1134193

**Published:** 2023-03-06

**Authors:** Sufang Fang, Yubo Shi, Peng Wang, Chen Guan, Xiaolong Gu, Lihui Guan, Ping Cui, Xun Suo

**Affiliations:** ^1^College of Animal Science and Technology, Hebei North University, Zhangjiakou, Hebei, China; ^2^Zhangjiakou Animal Husbandry Technology Promotion Station, Zhangjiakou, Hebei, China; ^3^National Key Laboratory of Veterinary Public Health Security, Key Laboratory of Animal Epidemiology and Zoonosis of Ministry of Agriculture, Beijing, China; ^4^National Animal Protozoa Laboratory and College of Veterinary Medicine, China Agricultural University, Beijing, China

**Keywords:** *Eimeria kongi*, pathogenicity, immunogenicity, endogenous development, drug sensitivity

## Abstract

Following the discovery of *Eimeria kongi*, we investigated the pathogenicity, immunogenicity, endogenous development and drug sensitivity of this coccidian. Coccidia-free rabbits were inoculated with 1 × 10^2^ to 5 × 10^4^ sporulated oocysts of *E. kongi* before challenge 14 days post inoculation. *E. kongi* was moderately pathogenic and induced good immunity against re-infection. All inoculated doses results in reduced food intake and body weight gain, and an inoculation oocyst dose of 1 × 10^3^ or higher caused various degrees of diarrhea. Except for one death of the highest dose group, all rabbits recovered 12 days post inoculation. An inoculation dose of 1 × 10^3^ or 1 × 10^4^ oocysts conferred the most effective protection from re-infection, which reduced oocyst output by approximately 99% and maintained body weight gain. Four generations of schizogony were observed, and the endogenous development mainly occurred in the jejunum and ileum of rabbits. *E. kongi* was most sensitive to sulfachloropyrazine sodium, followed by decoquinate; it is resistant to diclazuril. Both decoquinate and sulfachloropyrazine sodium may be effective in the control of *E. kongi* infection.

## 1. Introduction

It was recognized that the monoxenous coccidia of the genus *Eimeria* Schneider, 1875 (*Apicomplexa*: *Eimeriidae*) is a protozoan parasite, which can cause coccidiosis in animals including livestock and is mainly characterized by severe enteritis involving diarrhea (1, 2). The infection is transmitted by the fecal-oral route of the sporulated oocysts, and its life cycle includes three different phases, schizogony (proliferation of schizozoites in schizonts), gametogony (forming gametes, zygotes, and oocysts) in the hosts, and sporogony (maturation of the oocysts) in the external environment. The parasites possess narrow host spectra and each species of Eimeria displays different pathogenicity in their specific hosts (3, 4).

Eleven species of *Eimeria* have been identified as pathogens of rabbit coccidiosis (5), namely *E. stiedae, E. vejdovskyi, E. flavescens, E. media, E. intestinalis, E. perforans, E. piriformis, E. coecicola, E. exigue, E. magna, and E irresidua*. Coccidiosis can be classified into two forms: hepatic coccidiosis that occurs because of *E. stiedae* and intestinal coccidiosis that occurs because of the remaining members of Eimeria. it has been reported that parasites of Eimeria species can induce significant economic losses in the farm and pet industries due to high infection and mortality rates (6). Cui et al. conducted a survey on the infection rate of coccidia in rabbits in some areas of Hebei Province. The results showed that the infection rate of coccidia in rabbits was relatively high, with the average infection rate of 96.7% in young rabbits, 75.1% in adult young rabbits and 55.7% in breeding rabbits (7). In 2017, Cui et al. discovered a new species, *E. kongi*, in Zhangjiakou of Hebei, China. *E. kongi* was identified as a new rabbit coccidia species based on morphological characteristics, prepatent period and phylogenetic analyses of the 18S rDNA gene and the first internal transcribed spacer (ITS-1) (8). The aims of this paper was to study the pathogenicity, immunogenicity, endogenous development, and drug sensitivity of *E. kongi*.

## 2. Materials and methods

### 2.1. Test materials

#### 2.1.1. Coccidia strain

*E. kongi* was provided by the Animal Parasitology Laboratory of Hebei North University and was subcultured before our experiments (8).

#### 2.1.2. Rabbits

Weaned New Zealand white (NZW) rabbits (35-day-old) with similar body weights were obtained from a local rabbitry. They were housed in a coccidia-free environment with free access to water and in-house feed free of anticoccidial drugs. Feed and water were heated at 80°C for 2–3 h to kill any coccidia oocysts. The rabbits were confirmed as coccidia-free if there were no coccidian oocysts after three fecal examinations once every other day. Coccidia-free rabbits were selected for the experiments until approximately 45-day-old. Experimental rabbits were subjected to a bivalent vaccine against rabbit hemorrhagic disease virus (RHDV) and *Pasteralla multocida* at 37 and 62 days of age.

#### 2.1.3. Drugs

Decoquinate was purchased from Hubei Yunmei Technology Co., Ltd. (Wuhan, Hubei province, China), diclazuril from Hebei Pude Animal Pharmaceutical Co., Ltd. (Luquan, Shijiazhuang, Hebei province, China), and sulfachloropyrazine sodium from Hebei Zhongbei Jiamei Biotechnology Co., Ltd. (Zanhuang, Shijiazhuang, Hebei province, China). Xylazine Hydrochloride Injection from Dunhua Shengda Animal Pharmaceutical Co., Ltd. (Dunhua, Jilin province, China).

### 2.2. Experimental methods

#### 2.2.1. Pathogenicity and immunogenicity of *E. kongi*

Thirty coccidia-free rabbits of 45-day-old were randomly assigned to 6 groups of 5 rabbits each. They were inoculated with 1 × 10^2^ (group I), 1 × 10^3^ (group II), 1 × 10^4^ (group III), and 5 × 10^4^ (group IV) sporulated oocysts of *E. kongi*, respectively, as the immunized groups. One group (Group V) was unimmunized and challenge control (UCC) group, and another group (Group VI) was unimmunized and unchallenged control (UUC) group. Fourteen days post inoculation (DPI), all rabbits of Groups I–V were orally challenged with 1 × 10^4^ sporulated oocysts.

Water intake and feed consumption was recorded and feces was examined daily. The rabbits were weighed every 7 days within 14 DPI. Feces were collected and weighed 7–14 days post inoculation and 7 to 14 days post challenge (DPC), oocysts per gram of feces was measured by the McMaster method, and the total oocyst output of each group was calculated.

McMaster method: Two grams of feces were soaked for 2–5 min in 10 ml of water in mortar, the fecal slurry was then mixed with 50 ml of saturated sodium chloride solution and homogenized using a glass rod, and filtered through a metal sieve. Following agitation, the sample was drawn from the suspension using a pipette, and introduced into two chambers of a McMaster oocysts counting slide which was then placed on the stage of a compound light microscope. After 3 min, the number of oocysts in 1 cm^2^ of the chamber was counted, and the average of the two chambers was calculated and then multiplied by 200 to obtain the number of OPG.

#### 2.2.2. Endogenous development of *E. kongi*

To study the development of *E. kongi*, 39 coccidia-free rabbits of 45-day-old were inoculated with *E. kongi* oocysts at doses from 1 × 10^4^ to 2 × 10^7^, which was selected on the basis of the pathogenicity experiment, and then sacrificed at various time points for the observation of development stages of *E. kongi* (Table 1).

**Table 1:** Inoculation doses and time of sacrifice after infection.

**Animal number**	**Sampling time (h)**	**Inoculation dose**	**Animal number**	**Sampling time (h)**	**Inoculation dose**
1	12	2 × 10^7^	8	96	1 × 10^6^
2	24	2 × 10^7^	9	108	1 × 10^6^
3	36	2 × 10^7^	10	120	2 × 10^4^
4	48	1 × 10^7^	11	132	2 × 10^4^
5	60	1 × 10^7^	12	144	1 × 10^4^
6	72	5 × 10^6^	13	156	1 × 10^4^
7	84	2.5 × 10^6^			

Three rabbits at each time point after inoculation was euthanized by Xylazine Hydrochloride Injection (1 ml/kg body weight) into the ear vein. Duodenum, jejunum, ileum, cecum, colon, and rectum were collected aseptically and fixed in 10% formalin for 48 h, respectively. The collected tissues were then dehydrated, embedded in paraffin, and sectioned. The tissue sections were stained with hematoxylin and eosin (HE) and observed under a light microscope. Size of at least 30 schizonts were measured. 30 microscope fields were observed in each generation, and the number of type A schizont and type B schizont in each field was recorded, and the ratio of type A schizont to type B schizont was calculated.

#### 2.2.3. Drug sensitivity of *E. kongi*

Fourty rabbits of 45-day-old coccidia-free were divided into 5 groups with 8 rabbits per group. Group I was decoquinate treatment group, group II was sulfachloropyrazine sodium treatment group, group III was diclazuril treatment group, group IV was inoculated and untreated control group, and group V was uninoculated and untreated control group. Each rabbit in groups I–IV was inoculated with 1 × 10 4 oocysts. Three drugs were given separately the day before inoculation, decoquinate at a dose of 0.5 g/kg in feed and sulfachloropyrazine sodium at a dose of 1 g/L in drinking water were given for 8 consecutive days, and diclazuril was administered at a dose of 1 mg /L in drinking water for 5 consecutive days (Table 2). The animals were observed daily for food intake, water consumption and clinical signs, and body weight was measured every 7 days within 14 days post inoculation (DPI). Feces was collected daily and oocysts in feces were enumerated using the McMaster method from 7 to 14 days post inoculation (DPI).

**Table 2:** Experimental design of the drug sensitivity study.

**Group**	**Drug**	**Dosage and days of administration**	**Dose of oocysts inoculated/rabbit**
Group I	Decoquinate	0.5 g/kg in feed 8 days	1 × 10^4^
Group II	Sulfachloropyrazine sodium	1 g/L in drinking water 8 days	1 × 10^4^
Group III	Diclazuril	1 mg /L in drinking water 5 days	1 × 10^4^
Group IV	—	—	1 × 10^4^
Group V	—	—	—

### 2.3. Data analysis

The SPSS19.0 software was used for statistical analysis by analysis of variance and student's *t*-test. ^*^ indicates *P* < 0.05, ^**^ indicates *P* < 0.01, without a right superscript asterisk, indicates *P* > 0.05.

## 3. Results

### 3.1. Pathogenicity and immunogenicity of *E. kongi*

#### 3.1.1. Clinical signs

After inoculation oocysts of *E. kongi*, rabbits exhibited a decrease in appetite and had soft feces. These clinical signs appeared on the 3rd day after inoculation of 5 × 10^4^ oocysts, and on days 4–7 after the 1 × 10^2^, 1 × 10^3^ or 1 × 10^4^ inoculation dose. The rabbits in the highest dose group had very little food and were lethargic, and all had different degrees of diarrhea, and one rabbit had alternating episodes of constipation and diarrhea and died on day 8. In the 1 × 10^4^ group, 60% of the rabbits had diarrhea and in the 1 × 10^3^ group 20% had diarrhea. No diarrhea was seen in the lowest inoculation dose group (1 × 10^2^). On days 8–9, the rabbits in the 1 × 10^2^ and 1 × 10^3^ groups returned to normal, and rabbits of all groups appeared normal by day 12.

All groups except for the UUC group were challenged with 1 × 10^4^ oocysts 14 days after the inoculation. After the challenge, the rabbits of the 1 × 10^2^ − 1 × 10^4^ immunized groups developed milder clinical signs. The animals in the 1 × 10^2^ and 1 × 10^3^ immunized groups exhibited decreased appetite, while the rabbits in the 1 × 10^4^ immunization group did not consume any feed and were of poor condition on day 4, but none of the animals in these groups had soft feces or diarrhea. The highest immunization dose group (5 × 10^4^) consumed no feed on day 4 and 50% of them developed soft feces, and 25% had diarrhea. Starting from day 7, conditions of all rabbits of immunized challenged groups began to improve, and they all appeared normal by day 12. Clinical signs of the UCC group were similar to those observed after the immunization dose of 1 × 10^4^, with decreased feed intake and soft feces on day 4, and diarrhea in 60% rabbits on day 5–6. All rabbits of the UCC group appeared normal 12 days after challenge.

#### 3.1.2. Changes in body weight gain and oocyst output

**Figure 1:**
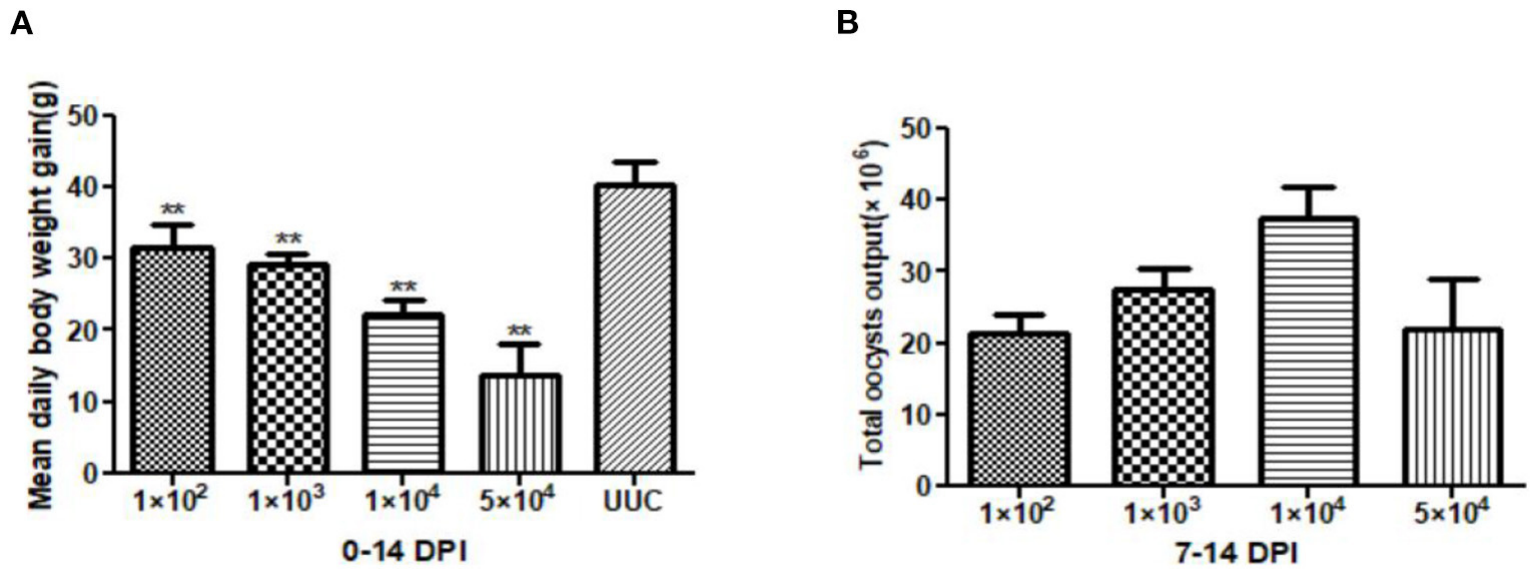
Average daily weight gain of rabbits and total oocyst output in pathogenicity tests. **(A)** Average daily weight gain of rabbits 0–14 days after initial oocyst inoculation. **(B)** Total oocyst output 7–14 days after inoculation with different doses of sporulated *E. kongi* oocysts. DPI: day post initial inoculation. **P < 0.01 (Student's *t*-test).

At 14 days post inoculation (DPI), the average daily weight gain of the rabbits in each immunized group was significantly different from that of the rabbits in the UUC group (*P* < 0.01) (Figure 1A), body weight gain with increasing inoculation doses was reduced by 21.71, 27.40, 44.84, and 65.84%, respectively.

The total oocyst output after immunization ranged from 2.13 × 10^7^ to 3.74 × 10^7^, with the 1 × 10^4^ dose group having the highest oocyst output and the 1 × 10^2^ dose group the lowest oocyst output (Figure 1B). Interestingly, total oocyst output of the highest inoculation dose (5 × 10^4^) was similar to the output of the 1 × 10^2^ dose group.

**Figure 2:**
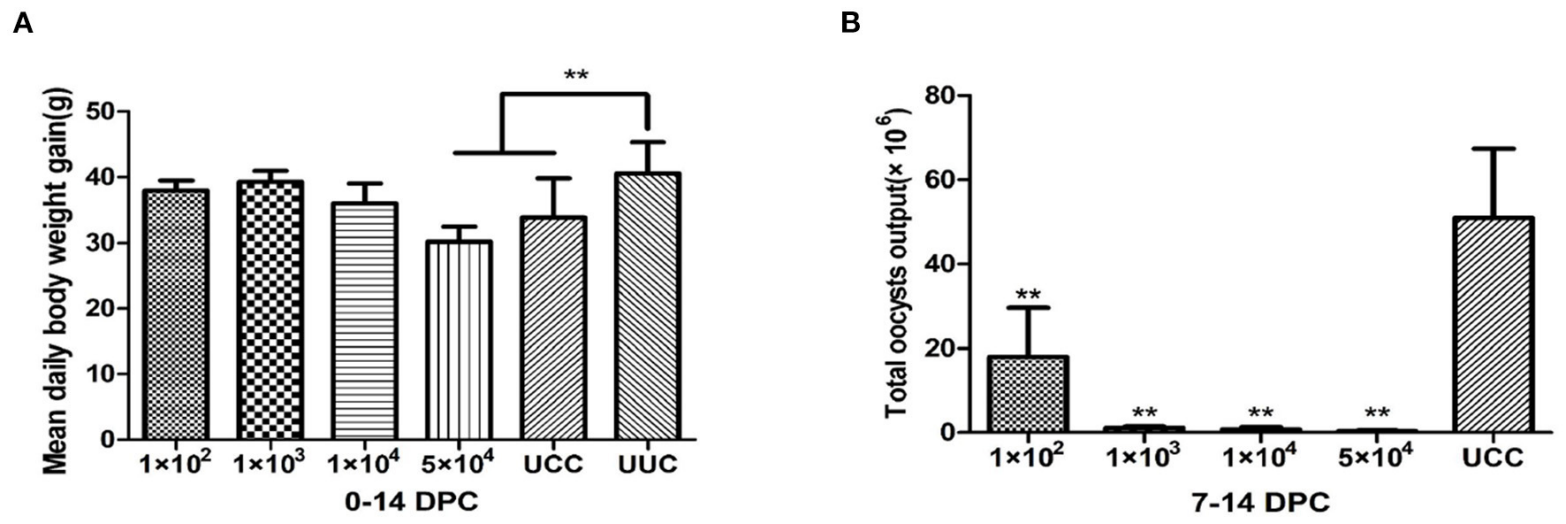
Average daily weight gain of rabbits and total oocyst output in immunogenicity tests. **(A)** Average daily weight gain of rabbits 0–14 days after challenge. **(B)** Total oocyst output 7–14 days after challenge with 1 × 10^4^ sporulated oocysts. DPC, day post challenge; UCC, unimmunized, challenged control group; UUC, unimmunized, unchallenged control group. **P < 0.01 (Student's *t*-test).

The average daily weight gain of the rabbits in the 1 × 10^2^, 1 × 10^3^, and 1 × 10^4^ immunized and challenged groups at 14 days post challenge (DPC) was not significantly different from that of rabbits in the UUC group (*P* > 0.05). The average daily weight gain of rabbits in the 5 × 10^4^ immunized challenge group was significantly lower than that of the UUC group (*P* < 0.01) (Figure 2A).

The total oocyst output of rabbits in all immunized and challenged groups was significantly lower than that of the UCC (*P* < 0.01) (Figure 2B). Among the immunized groups, the rabbits in the 1 × 10^2^ immunization dose group had the highest oocyst output (1.8 × 10^7^ oocysts), but the output was still 64.6% lower than that of the UCC group. The oocyst output of the other 3 immunized and challenged groups decreased by 97.7, 98.5, and 99.2% in the 1 × 10^3^, 1 × 10^4^ and 5 × 10^4^ groups, respectively, compared with the UCC group.

### 3.2. Endogenous development of *E. kongi*

The tissue sections of intestines of rabbit infected with parasites were observated by light microscope to study endogenous development of *E. kongi*. Development stage was observed within a parasitophorous vacuole mainly in the epithelium of villi of jejunum and ileum. Four generations schizonts and one gametogony were observed, with two types of schizonts in each generation, namely type A schizont and type B schizont, both of which were ellipsoid. Type A schizont formed short and round, stubby schizozoites, while type B schizont gave rise to slender schizozoites.

**Figure 3:**
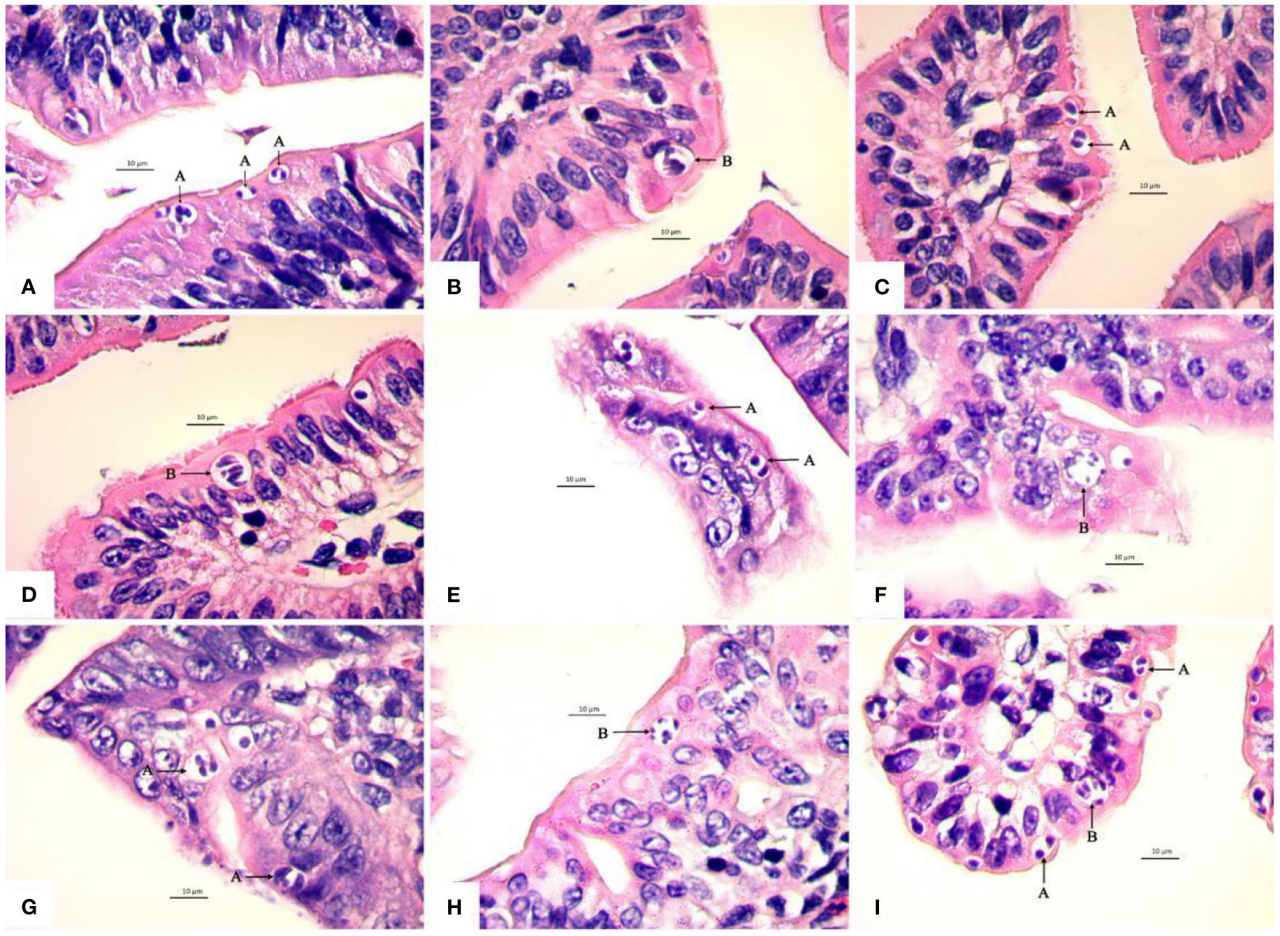
Light micrographs showing four schizogonies of *E. kongi* in the jejunum and ileum of experimentally infected rabbits (×1,000). Scale bar = 10 μm. **(A)** The type A schizonts (A) of the first generation at 72 h p.i. **(B)** The type B schizont (B) of the first generation at 72 h p.i. **(C)** The type A schizonts (A) of the second generation at 84 h p.i. **(D)** The type B schizont (B) of the second generation at 84 h p.i. **(E)** The type A schizonts (A) of the second generation at 96 h p.i. **(F)** The type B schizont (B) of the second generation at 96 h p.i. **(G)** The type A schizont (A) of the third generation at 108 h p.i. **(H)** The type B schizont (B) of the third generation at 108 h p.i. **(I)** The type A schizonts (A) and type B schizont (B) of the fourth generation at 120 h p.i.

The first generation schizonts were first seen 72 h post inoculation (p.i.) in the epithelium of villi and crypts of jejunum and ileum. The type A schizonts measured 6.42 (4.43–8.81) × 5.29 (3.41–6.99) μm, with 2–4 schizozoites (Figure 3A), whereas the type B schizonts measured on average 9.63 (7.86–10.92) × 8.25 (6.97–9.27) μm and gave rise to 5~15 slender schizozoites (Figure 3B). The ratio of the number of A to B schizonts was 5.86:1 (Table 3).

**Table 3:** A survey of the endogenous development of *Eimeria kongi*.

**Stage**	**Hours post inoculation**	**Localization**	**Number of schizozoites in a schizont**		**Ratio of A:B schizonts**

			**Type A schizont**	**Type B schizont**	
First generation	72	Epithelium of villi and crypts of the jejunum and ileum	2–4	5–15	5.86:1
Second generation	84–96	Upper and middle parts of villi of the jejunum and ileum	2–4	5–15	1.96:1
Third generation	108	Epithelium and propria lamina of the villi of the jejunum and ileum	1–4	5–18	2.66:1
Fourth generation	120	Epithelium and propria lamina of villi and crypts of the jejunum and ileum	1–4	5–20	3.53:1
Gamogony	From 132	Epithelium of villi of the jejunum and ileum	–	–	–

The second generation schizonts occurred in the upper and middle parts of jejunum and ileum villi and were seen 84–96 h p.i. The type A schizont measured 6.77 (4.23–10.29) × 5.43 (3.44–7.12) μm and gave rise to 2–4 schizozoites (Figures 3C, E), whereas type B schizonts measured 9.91 (7.61–14.24) × 8.11 (5.94–12.15) μm and were estimated to produce 5–15 elongated schizozoites (Figures 3D, F). The ratio of A:B schizonts was 1.96:1 (Table 3).

The third generation of schizogony was observed 108 h p.i., mainly developing in epithelium and propria lamina of the villi of the jejunum and ileum. The type A schizont measured 7.11 (4.93–9.82) × 5.64 (3.37–8.39) μm and harbored 1–4 schizozoites (Figure 3G), whereas type B schizonts measured 9.72 (7.96–11.98) × 7.73 (6.27–9.71) μm and contained 5–18 schizozoites (Figure 3H). The ratio of A:B schizonts was 2.66:1 (Table 3).

The fourth generation schizonts were noted 120 h p.i. and they developed in the epithelium and propria lamina of villi and crypts of jejunum and ileum. The type A schizont measured 7.16 (3.86–11.44) × 5.63 (3.36–9.56) μm had 1–4 schizozoites (Figure 3I), whereas the type B schizont measured 9.47 (7.27–11.36) × 8.20 (6.11–10.89) μm and contained 5–20 schizozoites (Figure 3I). The ratio of A:B schizonts was 3.53:1 (Table 3).

At 132 h p.i., *E. kongi* entered gametogony. Additional to schizonts, macrogamonts and microgamonts were observed in epithelium of villi of the jejunum and ileum.

**Figure 4:**
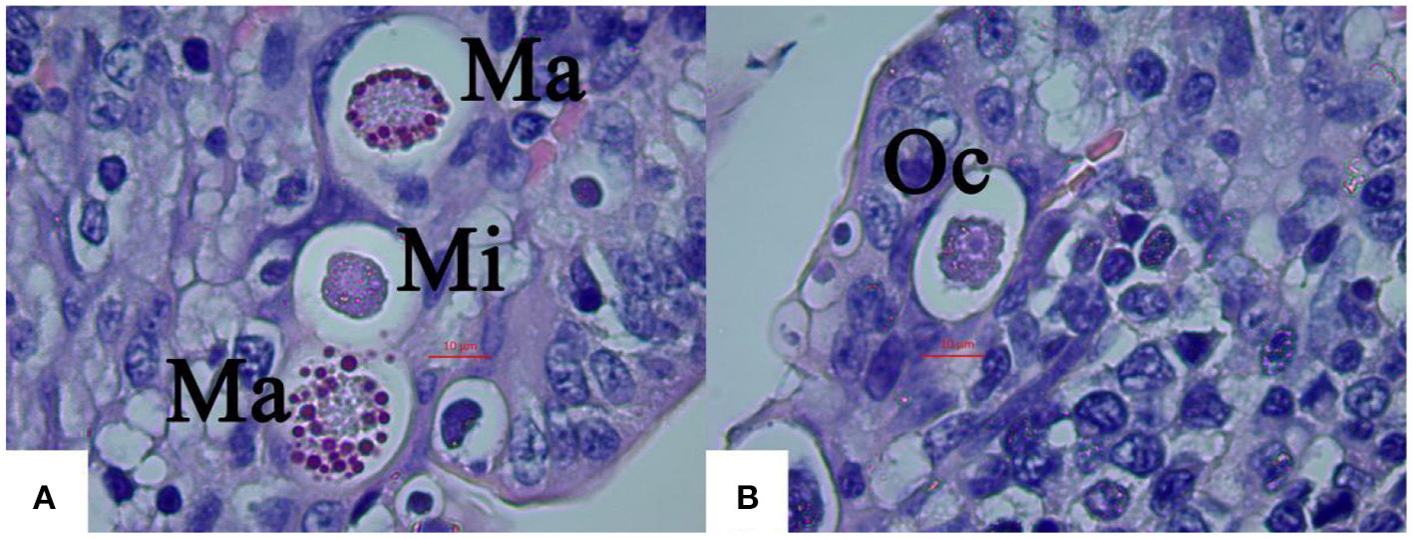
Light micrographs of gametogony of *E. kongi* experimentally infected rabbits (×1,000). Scale bar = 20 μm. **(A)** The microgamonts (Mi) and macrogamonts (Ma) of *E. kongi* in epithelial cells of ileal villi of rabbit at 132 h p.i. **(B)** Developing oocysts (Oc) at 144 h p.i.

The average size of macrogamonts was 17.48 × 16.31 μm (Figure 4A), and microgamonts 11.56 × 10.62 μm, both of which wre spherical or subspherical (Figure 4A). The protoplasm of microgamonts was darker and more concentrated than that of macrogamonts, forming a dark purple protoplasm, whereas macrogamonts were distinguishable from schizonts and microgamonts by their large central nucleus with a prominent nucleolus and many basophilic wall forming bodies at their periphery along the membrane.

Numerous oocysts at different stages of maturation could be distinguished at 144 h p.i, which was slightly ellipsoid (Figure 4B). Fully formed oocysts occurred in the feces of the infected rabbits 6 DPI.

### 3.3. Drug sensitivity of *E. kongi*

**Figure 5:**
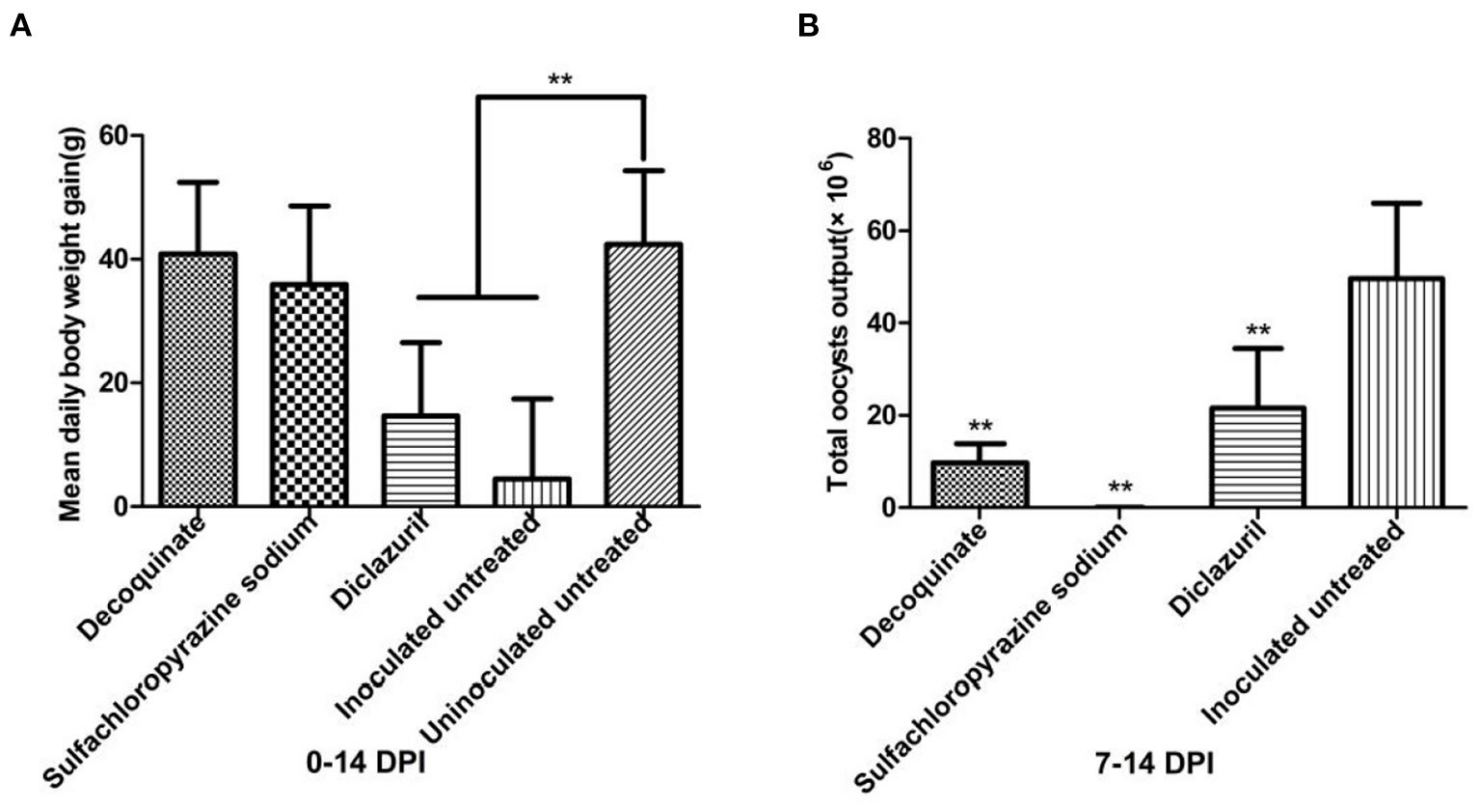
Comparison of mean daily body weight gain and total oocysts output in different groups. in drug sensitivity experiment of *E. kongi*. **(A)** Average daily weight gain. **(B)** Total oocyst output. DPI, day post inoculation. **P < 0.01 (Student's *t*-test).

*E. kongi*-inoculated rabbits treated with decoquinate or sulfachloropyrazine sodium gained significantly more weight than the inoculated and untreated rabbits 0–14 DPI (*P* < 0.01), and the body weight gain of the two treated groups was comparable with that of the uninoculated and untreated control group (*P* > 0.05) (Figure 5A). The decoquinate and sulfachloropyrazine sodium-treated rabbits also excreted significantly less oocysts than the inoculated and untreated control group rabbits 7–14 DPI (*P* < 0.01), and the two drugs inhibited oocyst output by 80.47 and 99.97%, respectively (Figure 5B).

The daily body weight gain of diclazuril-treated rabbits was significantly lower than that of the inoculated and untreated rabbits control group 0–14 DPI (*P* < 0.01) (Figure 5A). Diclazuril inhibited oocyst output by 56.61%, the total oocyst output of diclazuril-treated rabbits was significantly less than that of the inoculated and untreated group rabbits 7–14 DPI (*P* < 0.01) (Figure 5B). The diclazuril-treated rabbits and the inoculated and untreated group exhibited decreased appetite and had diarrhea, whereas the rabbits in the other experimental groups appeared normal.

## 4. Discussion

### 4.1. Pathogenicity and immunogenicity

The pathogenicity and immunogenicity of rabbit coccidia were evaluated based on clinical signs, weight gain and oocyst output (9–11). Zhangjiakou strain of *E. kongi* (*E. kongi*-ZJK) was determined to be moderately pathogenic according to the pathogenicity assessment criteria that rabbits caused by moderately pathogenic coccidia species showed symptoms of depression of growth, some cases of diarrhoea, mortality depending on the doses (12). In this study, except for rabbits in the 1 × 10^2^ infection group, rabbits in the other infection dose groups had different degrees of diarrhea and impaired weight gain. Diarrhea and decreased body weight gain were dose-dependent, but the total oocyst output was not linearly related to the inoculation dose. The total oocyst output of rabbits in the 1 × 10^4^ dose group was the highest, higher than that of the highest inoculation dose group (5 × 10^4^), which was most likely due to the crowding effect of coccidia, where fecundity decreases with increasing infective dose (13, 14). Pathogenicity can vary with Eimeria species and strains (12, 15). The pathogenicity of *E. kongi* is similar to that of *E. media, E. magna, E. irresidua, E. piriformis*, i.e., they have moderate pathogenicity.

In the highest dose group (5 × 10^4^), one rabbit died from coccidiosis 8 DPI, which was found to have lesions both the jejunum and ileum, but no lesions in other parts. The intestinal serosa was obviously hyperemia. The intestinal lumen was significantly expanded, and intestinal contents was thin, and a lot of mucus-like substance and the button-like necrosis were existed. The microscopy of scraping from the intestinal mucosa showed a large number of oocysts.

Weight gain and oocyst output after challenge infection as a criterion of immunogenicity be considered. Coudert et al. (9) showed that *E. intestinalis* is strongly immunogenic, In contrast, *E. flavescens* and *E. piriformis* (Coudert, pers. comm.) are weakly immunogenic. Other species, such as *E. irresidua, E. media, E. magna* could be considered as middle immunogenic (16). In this study, the immunization dose of 1 × 10^2^, 1 × 10^3^ and 1 × 10^4^ were most effective based on body weight gain, which was comparable with the weight gain of UUC group. Body weight gain of the 5 × 10^4^ immunized group was significantly lower than that of the UUC group, which may be related to the serious damage to intestinal tissues caused by the large immunization dose, proliferation of intestinal pathogenic bacteria, and synergistic effects of bacteria and coccidia oocysts of challenge. However, the highest immunization dose (5 × 10^4^), as well as the 1 × 10^3^ and 1 × 10^4^ immunization doses, almost completely suppressed oocyst excretion in feces, whereas the lowest immunization dose (1 × 10^2^) reduced oocyst output only by 65%. Based on body weight gain and oocyst output, *E. kongi* confers good immunogenicity, and an inoculation dose of 1 × 10^3^ – 1 × 10^4^ would provide the most effective protection from coccidia infection.

### 4.2. Endogenous development

Schizogony are generally classified to generations based on the localization and distribution of coccidian schizonts, the time when a large number of mature schizonts emerge, the size and number of schizozoites, and the ratio of A: B schizonts. Pakandl et al. (17). In this study, schizogony of *E. kongi* could be divided into 4 generations mainly based on the localization, distribution of coccidian schizonts and the ratio of A: B schizonts (Table 3). *E. intestinalis, E. magna, E. media* and *E. irresidua* are parasitic in the jejunum and ileum (18–21). Our research suggests that the endogenous development of *E. kongi* proceeds in the jejunum and ileum of rabbits.

For *E. kongi*, there was no difference in the size of type A schizont and type B schizont between different generations, nor in the number of schizozoites in type B schizont (Tables 3, 4). The number of type A schizonts was more than that of type B schizonts; the mean ratio of type A to type B schizonts ranged from 1.86 to 5.86. As the localization of schizont appearance and the ratio of type A schizont to type B schizont were consistent at 84 h p.i. and 96 h p.i., the second asexual generation was considered at 84–96 h p.i.

**Table 4:** A comparison of the endogenous development of *E. kongi* and *E. irresidua*.

**Stage**	***E. Kongi***			***E. irresidua*** **(****20****)**		
	**Hour post inoculation**	**Localization**	**Size of schizonts and gamonts (**μ**m)**	**Hour post inoculation**	**Localization**	**Size of schizonts and gamonts (**μ**m)**
First merogony	72	Epithelium of jejunum and ileum villi	Type A: 6.42 × 5.29 Type B: 9.63 × 8.25	72–108	Glands of jejunum and ileum	No data
Second merogony	84–96	Upper and middle regions of jejunum and ileum villi	Type A: 6.77 × 5.43 Type B: 9.91 × 8.11	96–144	Lamina propria of jejunum and ileum	35.7 × 30.7
Third merogony	108	Epithelium and lamina propria of jejunum and ileum villi	Type A: 7.11 × 5.64 Type B: 9.72 × 7.73	132–216	Epithelium of jejunum and ileum	22.8 × 20.7
Fourth merogony	120	Mucosal epithelium and lamina propria and jejunum and ileum crypts	Type A: 7.16 × 5.63 Type B: 9.47 × 8.20	132–216	Epithelium of jejunum and ileum	22.8 × 20.7
Gamogony	≥132	Epithelium of jejunum and ileum villi	Microgamont: 11.56 × 10.62 Macrogamont: 17.48 × 16.31	>163	Epithelium and lamina propria and jejunum and ileum	Microgamont: 37.2 × 32.6 Macrogamont: 33.9 × 20.5

Although *E. kongi* resembles *E. irresidua* in shape and structure, they differ significantly in size and shape index of oocyst and prepatent time (8). By comparison, both *E. kongi* and *E. irresidua* have four generations of schizogony, but there are significant differences in the location of asexual generation and gamogony, as well as the size of schizonts and gametophytes (Table 4).

Four generations of schizonts have also been reported for other coccidia species, such as *E. flavescens* by light microscopic examination of HE stained tissue sections Wang et al. (22), and *E. magna* by light and electron microscopic examination Pakandl et al. (19). However, 5 generations of schizogony of the same coccidia species, *E. flavescens* (17, 22) and *E. magna* were reported by Pakandl et al. (19) by electron or light microscopy. The classification of different number of schizont generations for the same species coccidia could be due to different study methods.

The endogenous development and generation division of *E. kongi* could be confirmed by transmission electron microscopy, which would also be able to show whether type A schizonts and type B schizonts contain polynucleate schizozoites and uninucleate schizozoites, respectively (16, 17, 19, 23, 24).

The endogenous development and division of asexual generations of *E. kongi* will be further determine by transmission electron microscopy, and whether type A schizonts and type B schizonts contain polynucleate and uninucleate schizozoites, respectively.

### 4.3. Drug sensitivity

Many factors may affect the sensitivity of parasites to drug treatment. Previous exposures to other drugs and long-term use of the same drug could induce drug resistance (25, 26). Coccidia in different regions or locations can also have different susceptibily to drug treatment (27, 28). Decoquinate has been demonstrated in in-house and university studies to be an active coccidiostat when fed daily at 0.5 mg/kg body weight during periods of oocyst exposure. In our study, treatment of *E. kongi* with 0.5 g/kg of decoquinate in feed was effective.

In a previous study in artificially infected rabbits (29), diclazuril was shown to be highly effective against *E. intestinalis, E. magna* and *E. perforans* in semi-field conditions (30). Diclazuril was also optimally effective at 1 ppm in the feed in the prevention and cure of intestinal and hepatic coccidiosis in rabbit (29). The superior efficacy of curative use of diclazuril and sulphachloropyrazine against rabbit coccidiosis was reported (31). In this study, we observed that *E. kongi* was resistant to the recommended clinical dose of diclazuril, and the rabbits experienced severe growth inhibition and oocyst discharge after treatment. Sulfachloropyrazine sodium was the most effective drug of 3 anticoccidial drugs tested in this study. Although both decoquinate and sulfachloropyrazine sodium effectively inhibited the reproduction of *E. kongi*, sulfachloropyrazine sodium inhibited oocyst output in feces by 99.97% compared with 80.47% inhibition by decoquinate. Infected rabbits treated with both drugs maintained normal growth performance. Both decoquinate and sulfachloropyrazine sodium were effective in the control of *E. kongi* infection.

## 5. Conclusion

*E. kongi* was moderately pathogenic and induced good immunity against re-infection. Four generations of schizogony were observed, and the endogenous development mainly occurred in the jejunum and ileum of rabbits. *Eimeria kongi* was most sensitive to sulfachloropyrazine sodium, followed by decoquinate; it is resistant to diclazuril. Both decoquinate and sulfachloropyrazine sodium were effective in the control of *Eimeria kongi* infection.

## Data availability statement

The original contributions presented in the study are included in the article/supplementary material, further inquiries can be directed to the corresponding authors.

## Ethics statement

The animal study was reviewed and approved by the specialized Ethics Committee of Hebei North University. Written informed consent was obtained from the owners for the participation of their animals in this study.

## Author contributions

PC and XS conceived and designed the experiments. SF, YS, and PW performed the study of pathogenicity, immunogenicity, and endogenous development. CG and XG tested the sensitivity of *Eimeria kongi* to three drugs. LG collected and analyzed data. SF and YS wrote the first manuscript. XS critically revised the manuscript. All authors have read and approved the final version of the manuscript.
